# The Hospital Crisis: How Not to Get Money

**Published:** 1919-11-22

**Authors:** 


// The Hospital

The Workers' Newspaper of Administrative Medicine and Institutional Life, Administration, National Insurance and Health.

No. 1746, Vol. LXVII. SATURDAY, NOVEMBER 22, 1919. PRICE TWOPENCE

### THE HOSPITAL CRISIS: HOW NOT TO GET MONEY.

There are certain factors in the hospital crisis which every one responsible for a voluntary hospital's finances and future success should have clearly before him. The first is to realise that old methods of raising money for voluntary hospitals and other institutions have had their day. Before the war it had come to be the system in most hospitals to depend upon relatively few people—having regard to the population which the institution served—for the financial support necessary to enable the hospital to provide the medical or surgical treatment required to meet the needs of the poorer inhabitants. In some cases a goodly number of those who received in-patient treatment were in reality capable of making a reasonable payment for the benefits they received, though custom and practice had created and sustained the feeling that the voluntary hospitals were charities and not, as they are in reality first of all, cure-houses for the inhabitants of a given area which they serve. Let each pay what he can. This cry of charity was overdone. It angered both those who sought hospital care and those who were canvassed for money to support the system, and in latter years in many places the income available was not always, if ever, adequate to supply the means to introduce new developments of importance or to foot the bill of hospital costs. This was not surprising, because those institutions which worked the charitable appeal idea hardest were not infrequently under-officered or without the driving force, up-to-date knowledge and quickened apprehension, in the absence of which no man or woman can be qualified to take an active part in the financial development of a great voluntary hospital.

Another defect of the system was that individuals attached themselves to a hospital, took an active and zealous part in raising money in its support, and, when successful, they had the temptation of seeking a reputation outside the hospital as beggars. Experience demonstrates this is a practice which invariably and increasingly, as the years pass, excites dissatisfaction in the minds of the largest givers who tire of the appeals continuously issued and widely circulated, with some stimulating literature of a more or less direct character. Resentment may thus be caused, seeing that of all the persons who should be thanked, and whose services should be acknowledged first, come those men and women who, from a desire to render personal service and utilise the means with which they are blessed, help the sick and suffering to the fullest possible extent, in the fields of treatment and cure that specially appeal to them as individuals. Such givers habitually and of set purpose give of their means in furtherance of their desire. Fifty years' experience, and we could give a great number of instances in proof of the fact, demonstrate, that the field of voluntary offerings, like all fields which become increasingly productive, needs periodically a change in the office of husbandman or woman, so that the personality and the personal advertisement of the individual may be kept within bounds, and only continued for a reasonable period. The most successful financial administrators are those who have no personal aim whatever, but only the vision to give freely of the best of their talents in the cause of the suffering and the sick, for a term when the hospital's needs may have been greatest, and the demand for almost superhuman efforts approaches the breaking point.

We have not a word to say against any individual who has devoted a number of years to a given charity, putting much energy and time into the work; but there comes a moment when the best of such workers can undoubtedy render the most essential service to the institution to which they have attached themselves, by spontaneously giving place to new men, new methods, and new blood. We incline to the feeling that it might be found an economy, as long as the voluntary system endures, for each institution to employ an artist to paint a portrait of the zealous beggar, at the end of, say, ten years' work, to hang in the board-room and be presented on his making way for a new financial worker, a succession of whom should serve an apprenticeship, and really give time on the finance committee, that one or some of them may qualify and in turn succeed to the chairmanship. We are quite sure that Presentation Day would be attended by large numbers of donors who would celebrate the occasion by further contributions of even larger amounts in some cases, and continue to help the hospital to which they had attached their interest with increased vigour and continuous devotion.

We may add that in fifty-two years' continuous hospital work we have made it a practice to fix a limit to the years of special activity in raising money for a particular charity. The effect has been invariably to call forth most appreciative co-operation by the large donors throughout their life, and to result in frequent consultation when they had special sums to distribute, long after the time when we had deliberately discontinued to be the direct means of the gifts to be sought for the hospital or institution. This is an inside view and experience. It is one which prevails not only in this country but, having visited during our lifetime all the principal hospitals and institutions in most parts of the world, we feel convinced as a result of our study and inquiries that it may be taken as universal. The most successful money-spinner within our life's experience, a gentleman who is now the president of his hospital, is beloved and trusted by wealthy people of high and low degree. He has always kept himself in the background, and the great results he has achieved are realised only by so few a number of people that he belongs to the splendid type of worker and strength mentioned in Ecclesiasticus as they who have no memorial. Could any man or woman have a higher ideal when his life's work is done than to reach this level of human greatness?

This is the fourth week in which it has been our privilege in these columns to drive home the facts relating to the policy of shaking heads, wringing hands, and circulating ill-founded utterances—truth demands an even stronger statement in regard to this remark—having as their object to seek relief from the rates. Fancy the shame of it! When all the time hospital people who know their business are beginning to realise that the voluntary hospital fields are still ripe for the harvest, and require nothing but ability and organisation to bring in all the funds that are required to place the voluntary hospitals on a sounder financial foundation, than they have ever yet attained in this country. **May we venture for the fourth time, once more to ask our colleagues in the lay press to lend a hand in circulating the truth about this Voluntary Hospital Crisis? Pressmen need hospitals, like other men.** An association of a lifetime with them makes us confident, that if they will only examine the facts, the newspapers will soon ring with the right note, and bring rescue and triumph to the great voluntary hospital system, which is the glory of our country and the pride of the world.

[Fifth article next week: "How to Get all the Money Required."]

